# LOCKED IN SUFFERING: UNLOCKING HOPE AND IMPROVING CARE FOR ELDER INMATES: A CALL FOR ACTION

**DOI:** 10.1093/geroni/igad104.1604

**Published:** 2023-12-21

**Authors:** Raya Kheirbek, Kenzie Latham-Mintus

## Abstract

Our program will focus on several key areas, including care for hospitalized inmates, the importance of providing comprehensive care for all prisoners as mandated by 8th amendment, and the importance of identifying inmates facing end of life who would benefit from compassionate release. We will begin with a discussion on the challenges faced by elder inmates and address their unique physical, psychosocial and spiritual needs. With a growing population of aging prisoners, it is critical that we examine the healthcare and social services required to ensure that they receive adequate care. Next, we will explore the differences in healthcare provision for inmates versus non-inmates who are hospitalized. The presentation will examine the acute exacerbation of chronic disease, ethical considerations and legal requirements involved in providing care to both populations. We will also discuss the importance of interdisciplinary care for incarcerated individuals, which requires collaboration among medical providers, mental health professionals, social workers, chaplains and other specialists. Our panel of experts will share their experiences and provide insights on how this approach can improve health outcomes and reduce recidivism rates after release . Finally, we will hear from advocates who are working to ensure that all prisoners receive adequate healthcare and social services, They will share strategies for improving access to care, reducing health disparities, and addressing the unique needs of incarcerated elders Our symposium provides a unique opportunity to explore and share insights on how we can work together to improve the health and wellbeing of incarcerated individuals.

